# Comparison of the effect of dexmedetomidine intrathecal injection and intravenous infusion on subarachnoid blockade during knee arthroscopy procedures: a randomized controlled trial

**DOI:** 10.1186/s12871-023-02401-9

**Published:** 2024-01-05

**Authors:** Shujiao Liu, Yaorui Sun, YeWen Wang, Chao Sun, Quanyi Zhang

**Affiliations:** Department of Anesthesiology, Binzhou Medical University Hospital, Binzhou, 256603 China

**Keywords:** Dexmedetomidine, Subarachnoid block, Knee arthroscopy

## Abstract

**Background:**

Comparison of whether intrathecal dexmedetomidine prolongs spinal anesthesia-associated sensorimotor blockade more than intravenous infusion during knee arthroscopy procedures performed under subarachnoid blockade.

**Methods:**

Ninety patients aged 18–75 years, ASA class I-II, who underwent knee arthroscopy between October 2022 and April 2023 were randomized into intrathecal、intravenous and control groups.Subjects received three modes of administration: an intrathecal group (2 ml of 1% ropivacaine + 1 ml of 5 μg dexmedetomidine, along with intravenous saline infusion), an intravenous group (intrathecal 2 ml of 1% ropivacaine + 1 ml of 0.9% saline, with dexmedetomidine pumped intravenously at a dose of 0.5 μg/kg/h), and a control group (intrathecal 2 ml of 1% ropivacaine + 1 ml of 0.9% saline, along with intravenous saline infusion). Total analgesic duration, duration of sensory and motor blockade, Ramsay sedation score, Visual Analogue Score (VAS) at different postoperative time points, and occurrence of adverse effects were recorded.

**Results:**

The total analgesia duration was significantly longer in the intrathecal group than in the intravenous and control groups (352.13 ± 51.70 min *VS* 273.47 ± 62.57 min *VS* 241.41 ± 59.22 min, *P* < 0.001).The onset of sensory block was shorter in the intrathecal group than in the intravenous and control groups (4 [3–4]min *VS* 5 [4–5]min *VS* 5 [4–5]min; *P* < 0.001);the onset of motor block was shorter in the intrathecal group than in the intravenous group and the control group (5 [4–5]min *VS* 5 [5–6]min *VS* 6[5.5–7]min; *P* < 0.001).Sedation scores were higher in the intravenous group than in the intrathecal and control groups (*P* < 0.001). At 5 h postoperatively, the VAS score in the intrathecal group was lower than that in the intravenous and control groups (*P* < 0.001). At 24 h postoperatively, the VAS score in the intrathecal group was lower than that in the control group (*P* < 0.001). In addition, the incidence of bradycardia was significantly higher in the intravenous group than in the intrathecal and control groups (30%, 6.5%, and 3.4%, respectively; *P* = 0.018, *P* = 0.007).

**Conclusions:**

Intrathecal administration of dexmedetomidine did prolong the total analgesia duration, as well as accelerate the onset of sensory-motor blockade compared with intravenous infusion, and did not result in any hemodynamic instability or other adverse events at the doses studied.

**Trial registration:**

This single-center, prospective, RCT has completed the registration of the Chinese Clinical Trial Center at 26/09/2023 with the registration number ChiCTR2300076170.

## Introduction

Osteoarthritis of the knee and degenerative meniscus are common in the general population, their prevalence increases with age and they are a common cause of pain and disability [1]. Arthroscopic surgery is undoubtedly the mainstay of treatment for meniscal, ligament and cartilage injuries in sports medicine [2]. Analgesia is an important part of knee arthroscopy surgery [3]. As one of the most common anesthetic techniques, subarachnoid block can be used as the anesthetic of choice for lower extremity surgery such as knee arthroscopy, both for surgical needs and patient comfort. Due to the short duration of knee arthroscopy and the disadvantages of epidural catheter insertion such as catheter migration, breakage, infection, and inadvertent entry into blood vessels and the subarachnoid space [4], many techniques have been proposed to prolong the duration of anesthesia, such as the administration of drugs intravenously or the addition of local anesthetic adjuvants [5]. Local anesthetic adjuvants have been clinically explored by many anesthesiologists for their ability to provide good perioperative analgesia and reduce local anesthetic concentrations, and more and more clinical studies on local anesthetic drug adjuvants, both opioid and non-opioid, have emerged. Dexmedetomidine is difficult to ignore by clinical researchers as an α2-adrenergic receptor agonist with benefits such as sedation and analgesia. Several meta-analyses [5, 6] have shown that intrathecal dexmedetomidine shortens the time to onset of sensory-motor blockade and prolongs the duration of sensory and motor blockade, but with little hemodynamic alteration. Thus dexmedetomidine shows superior potential as an adjuvant to local anesthetics. In addition, it has also been suggested [7–9] that intraoperative intravenous infusion of dexmedetomidine can reduce postoperative opioid analgesic dosage and decrease the risk of postoperative stress and postawakening adverse events. The advantages and disadvantages of these two different routes of administration are controversial. The primary objective of this study was to assess the effect of intrathecal dexmedetomidine versus intravenous dexmedetomidine infusion on the total duration of analgesia in patients undergoing subarachnoid blockade during knee arthroscopy procedures. The secondary objective was to assess the differences between the different routes of administration on the onset of sensory and motor blockade and on intraoperative and postoperative Ramsay sedation scores and complications.

## Methods

The study was approved by the Research Ethics Committee of the Affiliated Hospital of Binzhou Medical College (Approval No. KYLL-2022–84, May 11, 2022), and all study subjects signed a written informed consent form, and the trial was registered with the Chinese Clinical Trial Registry (ChiCTR) (www. chictr.org.cn) with registration number ChiCTR2300076170.

### Inclusion criteria

Patients aged 18–75 years with ASA classification I-II who underwent knee arthroscopy between September 2022 and August 2023 at our institution were selected. Exclusion criteria were obesity (body mass index [BMI] > 30 kg/m^2^), contraindications to subarachnoid block, history of allergy to local anesthetics or dexmedetomidine, prolonged use of analgesic or sedative medications, bradycardia, failure of block, or refusal to undergo subarachnoid block. Using the G*Power software, the Effect size f was set to 0.35, We estimated that a sample size of 28 per group would provide 80% efficacy (α ≤ 0.05) to test for differences in duration of analgesia between the three groups.

### Study protocol

Patients were fasted for at least 8 h preoperatively, no other medications were given, and all patients were admitted to the operating room with open peripheral venous access and 10 ml/kg/hr of lactated Ringer's solution. Basic monitors were attached to the patient including pulse oximetry, ECG and NIBP. Oxygen was supplied to the patient via an oxygen mask,set at 4L/min. Patients were first randomized into intrathecal, intravenous, and control groups using computer-generated random number software. Where the anesthesiologist was unaware of the group assignment and the patient was unaware of the drug regimen received. A researcher who was not involved in subsequent anesthesia operations and data collection prepared the experimental solutions according to group assignment. The experimental reserve volume of the intrathecal group: dilute one unit of dexmedetomidine (specification: 2 mL, 200ug) to 40 ml, and extract 1 ml is 5ug; the experimental reserve volume of the intravenous group: take one unit of dexmedetomidine and dilute it to 50 ml, and install the syringe containing 50 ml of dexmedetomidine to a micro syringe pump, and infuse it at the speed of 0.5 μg/kg/h; regard the infused saline of the blank group as dexmedetomidine, and also infuse it according to the same speed, and convert it to ML, which is 0.125 ml/kg/h, which can achieve the same infusion volume as intravenous infusion of dexmedetomidine. In the intrathecal group, 2.0 ml (1%, 10 mg/ml) of ropivacaine + 1.0 ml (5 μg/ml) of dexmedetomidine was added to the local anesthetic solution pushed into the subarachnoid space, and the same infusion volume of saline as that of intravenous dexmedetomidine was infused intravenously while pushing in the local anesthetic solution, and the pumping was stopped at the beginning of the suture. In the intravenous group, 2.0 ml (1%, 10 mg/ml) of ropivacaine + 1.0 ml of 0.9% saline was added to the local anesthetic solution pushed into the subarachnoid space, while dexmedetomidine was infused intravenously at a rate of 0.5 μg/kg/h until the infusion was stopped at the beginning of the suture; In the control group, 2.0 ml (1%, 10 mg/ml) of ropivacaine + 1.0 ml of 0.9% saline was added to the local anesthetic solution pushed into the subarachnoid space, and the same infusion volume of saline as that of intravenous dexmedetomidine was infused intravenously at the same time as local anesthetic solution was pushed in, and the pumping was discontinued at the beginning of the suture. All patients underwent subarachnoid block in lateral position under aseptic precautions. It was performed with a 25G Quincke needle via a median approach in the L3-L4 gap using a standard midline approach. After clear cerebrospinal fluid was observed, 3 ml of configured local anesthetic solution was injected into the subarachnoid space at a rate of 0.1 ml/s, and the time of the end of the push of lumbar anesthetic solution was recorded. Then, the patient took the supine position. The degree of sensory block was assessed by using the pinprick technique every 1 min after the subarachnoid block to evaluate the sensory block until the time was recorded when the T10 level was reached, and the degree of motor block was assessed by using the modified Bromage scale, which was assessed every 1 min after the subarachnoid block (0 = no motor nerve block; 1 = unable to lift the leg; 2 = unable to bend the knee; 3 = unable to bend the ankle) until Bromage 3 was reached when time was recorded. The first monitoring of blood pressure and heart rate was the average of three consecutive measurements taken in the supine position when the patient arrived in the operating room and was defined as the baseline values.The second recording started with the local anesthetic pushed into the subarachnoid space, and systolic, diastolic and mean arterial pressures, heart rate and pulse oximetry were recorded, and thereafter at five-minute intervals until the end of the procedure.

Ramsay sedation scores were recorded at one-hour intervals until one hour postoperatively (1: patient anxious and restless; 2: patient cooperative, oriented, and quiet; 3: patient responsive to commands; 4: responsive to a light snap of the eyebrows or a loud auditory stimuli; 5: unresponsive to light brow snapping or loud auditory stimuli; and 6: no response), side effects such as bradycardia, hypotension, nausea, vomiting, and hypoxemia (SpO2 < 90%) were documented and appropriately managed. Hypotension was defined as SBP < 90 mmHg or > 30% decrease from baseline, and bradycardia was defined as HR < 50 beats/min. Hypotension was managed appropriately according to the situation, with 3 mg of ephedrine given intravenously if necessary, 0.4 mg of atropine given intravenously if necessary in the presence of bradycardia, and antiemetic ondansetron and oxygenation given appropriately in the presence of intraoperative nausea and vomiting, as well as hypoxemia. Postoperative pain scores were assessed using a Visual Analogue Scale (0 = no pain; 10 = worst pain) at 1 h, 5 h, and 24 h postoperatively. All patients were given an intravenous self-controlled analgesic pump (PCA): 1.5 μg/kg sufentanil plus saline configured to a total of 100 ml, with a first infusion of 0, a background infusion rate of 2 ml, and a lock-in time of 15 min; the time at which the subject first felt a painful stimulus to turn on the analgesic pump was recorded, and the total analgesic duration was defined as the time from the beginning of the end of the push of local anesthetic solution to the time of switching on the analgesic pump.

### Statistical analysis

All data were statistically analyzed using IBM SPSS Statistics for Mac version 27, firstly, the Shapiro–Wilk test was used to test the normal distribution of the continuous variables in the quantitative data, and the variables conforming to the normal distribution were expressed as the mean ± standard deviation, and if they did not conform to the normal distribution, they were expressed as the median (Quartile spacing), and qualitative data were expressed as numbers (proportions). Comparisons of continuous variables in quantitative data were analyzed using one-way ANOVA or Kruskal–Wallis test, and categorical data were analyzed using the χ2 test or Fisher exact test using Bonferroni-corrected significance levels for two-by-two comparisons. *p* < 0.05 was considered statistically different.

## Results

**Fig. 1:**
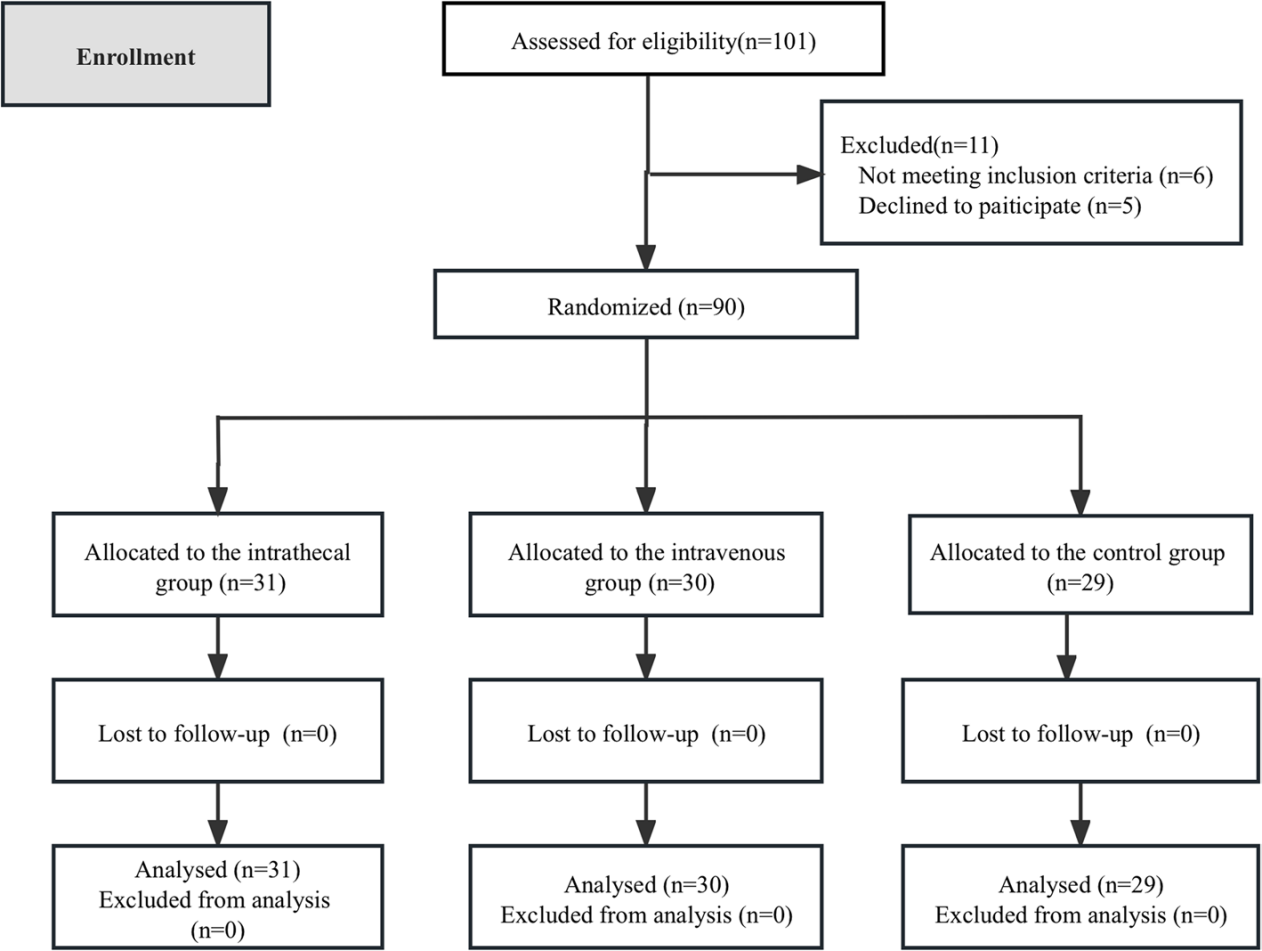
Flow diagram of study

Considering the loss of visits, a total of 101 patients were recruited, of which 6 did not meet the inclusion criteria for this study and 5 refused to participate in this study.Finally, 90 patients were included in this study and final analysis was performed, of which a total of 31 were included in the intrathecal group, 30 in the pump injection group, and 29 in the blank group (Fig. 1).

Demographic data are shown in Table 1.

**Table 1:** Demographic and perioperative characteristics

	intrathecal group(*n* = 31)	intravenous group(*n* = 30)	control group(*n* = 29)	P
Age(years)	48.9 ± 12.33	50.73 ± 15.12	49.45 ± 12.68	0.862^*,**,#^
Height(cm)	165.87 ± 10.79	165.23 ± 8.85	165.72 ± 8.88	0.964^*,**,#^
Weight(kg)	69(59–82)	70(65–75)	73(65–79)	0.445^*,**,#^
BMI(kg/m^2^)	25.25 ± 2.90	25.90 ± 2.88	26.16 ± 2.98	0.46^*,**,#^
male n (%)	14(45.16)	10(33.33)	15(51.72)	0.351^*,**,#^
ASA grade n (%)				0.636^*,**,#^
1	22(70.97)	18(60.00)	18(62.07)	
2	9(29.03)	12(40.00)	11(37.93)	
Baseline heart rate(times/min)	74.29 ± 9.10	77.20 ± 11.41	73.69 ± 11.91	0.413^*,**,#^
Baseline MAP(mmHg)	100.32 ± 8.48	103.08 ± 13.26	106.09 ± 11.83	0.15^*,**,#^
Duration of surgery(min)	70(46–105)	77(47.75–104.25)	59(41–110)	0.813^*,**,#^
Type of surgery				0.754^*,**,#^
Meniscus plasty	23	23	25	
Cyst removal	3	1	0	
Anterior cruciate ligament reconstruction	4	5	3	
Synoviectomy	1	1	1	

Data are expressed as mean ± SD, median (interquartile range), or number of the patients(proportion)

Abbreviations: *BMI*, body mass index; *ASA*, american society of anesthesiologist; *HR*, heart rate; *MAP*, mean arterial pressure

^*^intrathecal group versus intravenous group

^**^intrathecal group versus control group

^#^intravenous group versus control group

*P* < 0.05 considered as significant

The differences in demographic data, duration of surgery, and type of surgery among the three groups were not statistically significant (*P* > 0.05).

**Fig. 2:**
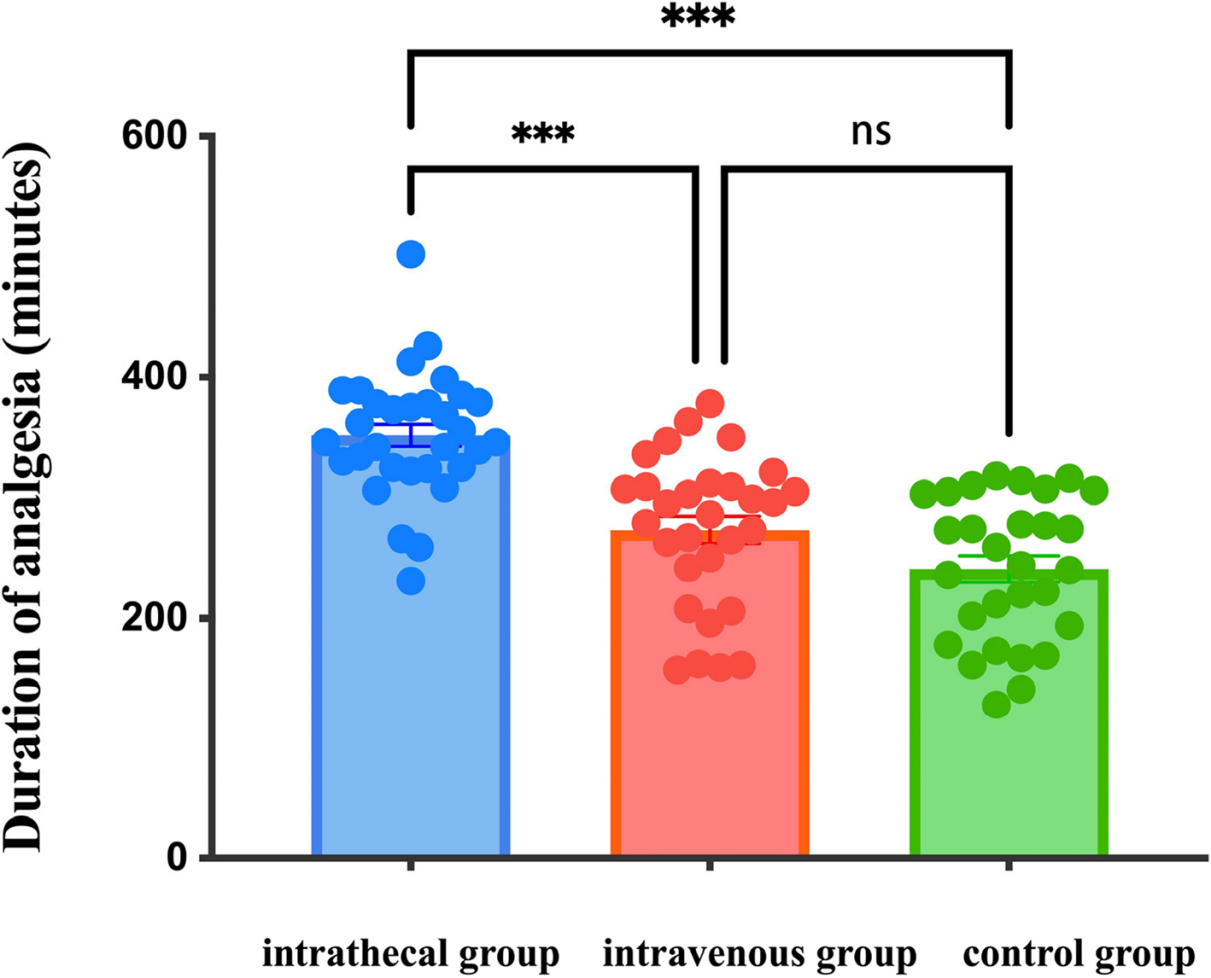
Total analgesic time between the three groups. Data are presented as bar graphs with mean (height of bar), mean ± standard deviation (top and bottom solid lines), and duration of analgesia for individual samples (solid dots). *** indicates *P* < 0.001; ns means the difference is not statistically significant

The main result was the total analgesic time between the three groups as shown in Fig. 2, the analgesic time in the intrathecal group (352.13 ± 51.70)min was significantly longer than that in the intravenous group (273.47 ± 62.57)min and the control group (241.41 ± 59.22)min; *P* < 0.001. There was no statistical significance in the difference between the intravenous group and the control group, *P* = 0.109 (Table 2).

**Table 2:** Sensory-motor block characteristics, analgesia and sedation between the three groups

	intrathecal group(*n* = 31)	intravenous group(*n* = 30)	control group(*n* = 29)	*P*
Time to reach T10 sensory block(min)	4(3–4)	5(4–5)	5(4–5)	< 0.001^*^, < 0.001^**^,1.0^#^
Time to reach Bromage3(min)	5(4–5)	5(5–6)	6(5.5–7)	< 0.001^*^, < 0.001^**^,0.384^#^
Total analgesic time(min)	352.13 ± 51.70	273.47 ± 62.57	241.41 ± 59.22	< 0.001^*^, < 0.001^**^,0.109^#^
Ramsay sedation score				
1 h after anesthesia	2(2–2)	3(3–3)	2(2–2)	< 0.001^*^,0.75^**^, < 0.001^#^
2 h after anesthesia	2(2–2)	2.25(2–3)	2(2–2)	< 0.001^*^,1.0^**^, < 0.001^#^
1 h after surgery	2(2–2)	2(2–3)	2(2–2)	< 0.001^*^,1.0^**^, < 0.001^#^
Visual Analogue Scale				
1 h after surgery	0(0–0)	0(0–0)	0(0–0)	0.093
5 h after surgery	1(0–2)	2.25(2–3)	3(2–3.25)	< 0.001^*^, < 0.001^**^,0.681^#^
24 h after surgery	2(1–2)	2(2–3)	3(2–3)	0.066^*^, < 0.001^**^,0.181^#^

^*^intrathecal group versus intravenous group

^**^intrathecal group versus control group

^#^intravenous group versus control group

*P* < 0.05 considered as significant

In addition, according to the Kruskal–Wallis test, the time required to reach the T10 sensory block plane was shorter in the intrathecal group (4 [3, 4] min) than in the intravenous group (5 [4, 5] min) and in the control group (5 [4, 5] min); *P* < 0.001. The time required to reach the Bromage 3 in the intrathecal group (5 [4, 5] min) was shorter than that in the intravenous group (5 [5, 6] min) and in the control group (6 [5.5–7] min); *P* < 0.001. Among them, there was no statistically significant difference between the intravenous and control groups for the time to reach the level of T10 sensory block and the time to reach Bromage 3 (*P* = 1.0; *P* = 0.384) (Table 2).

**Fig. 3:**
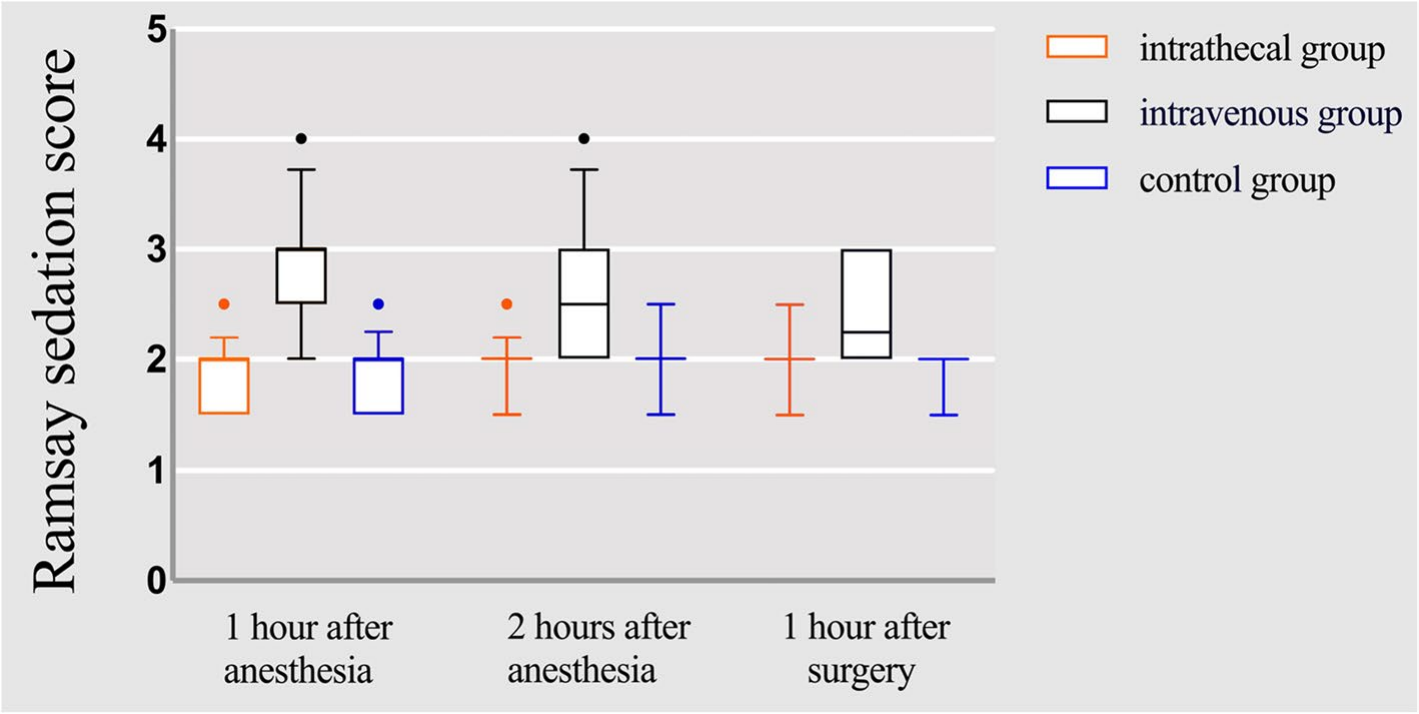
Ramsay sedation scores at 1 h after anesthesia, 2 h after anesthesia, and 1 h postoperatively between the three groups. Data are presented in box plots with ranges (top and bottom solid lines), interquartile spacing (boxes), medians (middle solid line), and outliers (solid dots)

Sedation scores are shown in Fig. 3, Ramsay sedation scores were higher in the intravenous group than in the intrathecal group and the control group at 1 h, 2 h after anesthesia and 1 h after surgery, and the difference was statistically significant (*P* < 0.001), whereas there was no statistically significant difference between the intrathecal group and the control group at any of the three time points (*P* = 0.75,*P* = 1.0,*P* = 1.0) (Table 2).

**Fig. 4:**
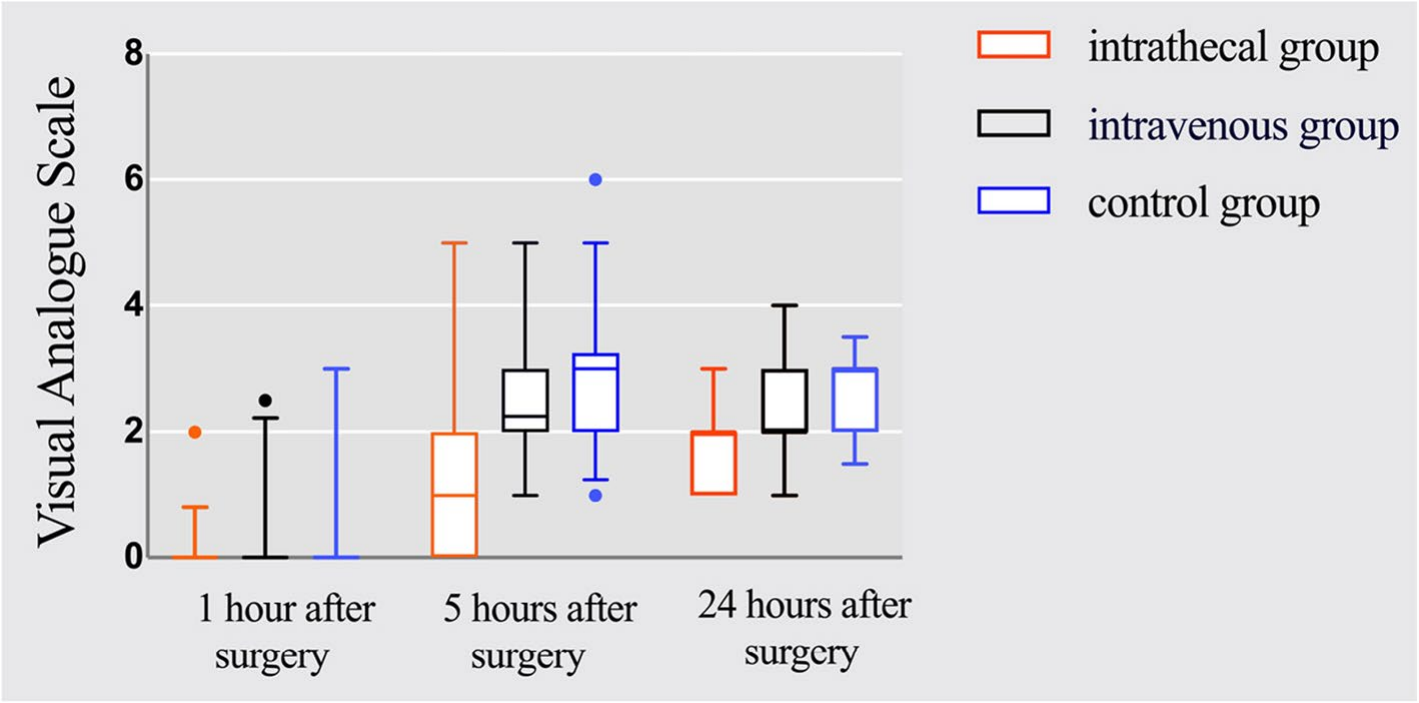
Visual analog scale (VAS) at 1, 5 and 24 h postoperatively between the three groups

Pain scores are shown in Fig. 4, and there was no statistical difference in VAS scores among the three groups at 1 h postoperatively (*P* = 0.093). At 5 h postoperatively, the VAS scores of the intrathecal group were lower than those of the intravenous group and the control group, and the difference was statistically significant (*P* < 0.001). At 24 h postoperatively, the VAS scores of the intrathecal group were lower than those of the control group (*P* < 0.001), and the difference in analgesic scores between the intrathecal group and the intravenous group was not statistically significant (*P* = 0.066). Among them, there was no statistically significant difference between the VAS scores of the intravenous group and the control group at 5 and 24 h after the operation (*P* = 0.681,*P* = 0.181).

No patient experienced hypotension SBP < 90 mmHg or > 30% decrease from baseline throughout the procedure. Bradycardia occurred in 9 patients in the intravenous group, 2 in the intrathecal group, and 1 in the control group, with rates of 30%, 6.5%, and 3.4%, respectively, a statistically significant difference (*P* = 0.018,*P* = 0.007), and no patient was treated with atropine. Only two cases of hypoxemia were observed in the intravenous group, and the difference between the three groups was not statistically significant (*P* = 0.21). In terms of nausea and vomiting, there was no significant difference between the groups (Table 3).

**Table 3:** Table adverse effects of anesthesia

	intrathecal group(*n* = 31)	intravenous group(*n* = 30)	control group(*n* = 29)	*P*
Bradycardia	2	9	1	0.018^*^,0.525^**^,0.007^#^
Hypotension	-	-	-	
Nausea or vomiting	1	1	1	1^*,**,#^
Hypoxaemia	0	2	0	0.21^*,**,#^

^*^intrathecal group versus intravenous group

^**^intrathecal group versus control group

^#^intravenous group versus control group

*P* < 0.05 considered as significant

## Discussion

In this trial, we conducted a study to determine what type of dexmedetomidine application is more effective in prolonging the total duration of analgesia during knee arthroscopy, thereby eliminating the possible complications associated with epidural catheter insertion as well as minimizing the physiological and psychological effects of the procedure on the patient and improving patient satisfaction. In this study, we designed a control group to compare intrathecal versus intravenous infusion, which demonstrated the extent to which the duration of analgesia was prolonged and the duration of sensory-motor block was shortened, as well as better reflecting the perioperative hemodynamic changes. A Meta-study [10] included 7 trials with a total of 364 patients eligible for analysis and investigated the effect of intravenous dexmedetomidine on spinal anesthesia compared to controls.This Meta-results showed that intravenous dexmedetomidine prolonged the duration of sensory blockade and delayed the time to the first request for analgesia after spinal anesthesia in spinal anesthesia patients. However, the results of our experiment confirmed that there was no statistical difference in the duration of analgesia with intravenous dexmedetomidine compared to the control group, However, based purely on the results of the study, it is clear that we can find that the duration of analgesia is indeed prolonged by intravenous dexmedetomidine infusion compared to the control group, which is not dependent on statistical analysis and does tend to objectively show some clinical benefit, on the contrary, if one relies purely on statistical analysis, since the analysis of this study was performed post hoc, the results here should be considered exploratory and intended to provide guidance for further deterministic studies. Therefore the choice of two-by-two comparisons in one-way analysis of variance (ANOVA) favored the use of the Bonferroni method, which is more conservative than the LSD method and did not yield statistically different outcome indicators. We therefore hope that clinical investigators will think in terms of the results of their studies and not be limited to the conclusions drawn by statistical methods.

Regarding the onset of sensory-motor blockade, the intrathecal group was significantly shorter than the intravenous group, contrary to the results of some studies [11], and the difference between the intravenous group and the control group was not significant, which was related to the long onset of action of intravenously administered dexmedetomidine, which is usually administered intravenously at 1.0 μg/kg of dexmedetomidine as a loading dose over a 10-min period in most of the experimental studies [7, 12], as it was reported [13], that heart rate can decrease by 30% from baseline up to 30 beats/min after an initial dose of 0.5 μg/kg administered over 10 min. The same high incidence of bradycardia was seen in the pretest before the start of the present experiment, and dexmedetomidine induced hemodynamic changes, especially after the loading dose.Therefore, in the present study, we did not set a loading dose, and only a maintenance dose of 0.5 μg/kg/h was set in this experiment, and dexmedetomidine was pumped intravenously during the push of local anesthetic solution from the subarachnoid space until it was stopped at the beginning of the suture, and therefore the lack of a loading dose characterization may bias the time of onset of sensory-motor block in the results of the study.Dexmedetomidine is a highly selective and specific α2-adrenergic receptor agonist.Hypotension and bradycardia are the most significant side effects of dexmedetomidine use; the results of the present trial showed that intrathecal dexmedetomidine did not increase the risk of hypotension and bradycardia. There was a greater reduction in heart rate in the intravenous group during the procedure compared to the control group, but no patient had received atropine; furthermore, there were two cases of hypoxemia in the intravenous group, and there was no statistical difference between the three groups in this regard. The intrathecal route appears to provide better postoperative analgesia, more stable hemodynamics, and fewer overall side effects.

The results of this study showed that the sedation scores at the three time points were significantly higher in the intravenous group compared to the other two groups, and the intrathecal group did not show superior sedation scores to the intravenous group in the postoperative period, which can be hypothesized to be due to an inconsistency in the pathway of sedation between the intrathecal route and the intravenous route. Related studies have shown [14] that the sedative effect of dexmedetomidine may be produced by inhibiting the release of norepinephrine from the hypothalamus of the brain, which ultimately leads to a decrease in histamine release, resulting in a sedative-hypnotic effect. The mechanism of analgesia for intravenous and intrathecal administration of dexmedetomidine seems to have different findings. Regarding the analgesic mechanism of the intravenous route, it has been suggested [15] that dexmedetomidine inhibits the release of norepinephrine through the activation of α2-adrenergic receptors, which reduces the sympathetic activity and produces analgesia; as for the analgesic mechanism of intrathecal injection, it has been suggested [16, 17] that the use of dexmedetomidine produces analgesia through the reduction of activation of ERK1/2 in the spinal cord by destructive solutions. The main problem facing the widespread clinical use of intrathecal dexmedetomidine is its potential neurotoxicity, but the vast majority of animal and human studies [18–20] have shown that intrathecal dexmedetomidine does not produce any neurologic dysfunction. Intrathecal dexmedetomidine may be an effective adjunct to a multimodal analgesic regimen for patients requiring subarachnoid blocks during knee arthroscopy. Of course, many of these studies are still in their preliminary stages, and the optimal intrathecal dose of dexmedetomidine and its long-term effects on neurologic function need to be explored in additional clinical trials.

There are some limitations of this article, firstly, we recorded the time when the patient first felt the pain stimulus to turn on the analgesic pump, therefore the total analgesic time is more dependent on the patient's subjective mood and there is no exact criterion, therefore a sufficiently large sample size is needed to eliminate systematic errors, in addition some objective indicators should be collected, for example, the stress response to pain produces many mediators at the molecular level, including inflammatory factors such as interleukin cytokines;Secondly, any drug has its safe dosage range, and the safe dosage ranges of different routes of administration of the same drug are even more different. Currently, the dose of intrathecal dexmedetomidine in clinical trials is mostly 5 μg [21], and this study did not set up any other dosage groups, failing to investigate whether the effect of dexmedetomidine given in the intrathecal way on outcome indicators was characterized by a dose-dependence.

In conclusion, intrathecal injection of 5 μg dexmedetomidine as an adjunct to local anesthesia prolonged total postoperative analgesia and shortened the onset of sensory and motor blockade without any hemodynamic instability or other adverse events.

## Data Availability

The datasets used and/or analysed during the current study are available from the corresponding author on reasonable request.

## References

[1] Beaufils P, Becker R, Kopf S (2017). Surgical management of degenerative meniscus lesions: the 2016 ESSKA meniscus consensus. Knee Surg Sports Traumatol Arthrosc.

[2] Redfern J, Burks R (2009). 2009 survey results: surgeon practice patterns regarding arthroscopic surgery. Arthroscopy.

[3] Kamdar PM, Liddy N, Antonacci C (2021). Opioid Consumption After Knee Arthroscopy. Arthroscopy.

[4] Maronge L, Bogod D (2018). Complications in obstetric anaesthesia. Anaesthesia.

[5] Xiong C, Han C, Lv H (2022). Comparison of adjuvant pharmaceuticals for caudal block in pediatric lower abdominal and urological surgeries: A network meta-analysis. J Clin Anesth.

[6] Paramasivan A, Lopez-Olivo MA, Foong TW, Tan YW, Yap APA (2020). Intrathecal dexmedetomidine and postoperative pain: A systematic review and meta-analysis of randomized controlled trials. Eur J Pain.

[7] Shin HJ, Do SH, Lee JS, Kim TK, Na HS (2019). Comparison of Intraoperative Sedation With Dexmedetomidine Versus Propofol on Acute Postoperative Pain in Total Knee Arthroplasty Under Spinal Anesthesia: A Randomized Trial. Anesth Analg.

[8] Kumari R, Kumar A, Kumar S, Singh R (2017). Intravenous dexmedetomidine as an adjunct to subarachnoid block: A simple effective method of better perioperative efficacy. J Anaesthesiol Clin Pharmacol.

[9] Ge DJ, Qi B, Tang G, Li JY (2016). Intraoperative Dexmedetomidine Promotes Postoperative Analgesia and Recovery in Patients after Abdominal Hysterectomy: a Double-Blind. Randomized Clinical Trial. Sci Rep..

[10] Abdallah FW, Abrishami A, Brull R (2013). The facilitatory effects of intravenous dexmedetomidine on the duration of spinal anesthesia: a systematic review and meta-analysis. Anesth Analg.

[11] Tang Y, Yang M, Fu F, Huang X, Feng Y, Chen X (2020). Comparison of the ED50 of intrathecal hyperbaric ropivacaine co-administered with or without intrathecal dexmedetomidine for cesarean section: A prospective, double-blinded, randomized dose-response trial using up-down sequential allocation method. J Clin Anesth.

[12] Liu X, Li Y, Kang L, Wang Q (2021). Recent Advances in the Clinical Value and Potential of Dexmedetomidine. J Inflamm Res..

[13] Sinha R, Kumar KR (2019). Intravenous dexmedetomidine augments the oculocardiac reflex. J AAPOS.

[14] Carollo DS, Nossaman BD, Ramadhyani U (2008). Dexmedetomidine: a review of clinical applications. Curr Opin Anaesthesiol.

[15] Iirola T, Aantaa R, Laitio R (2011). Pharmacokinetics of prolonged infusion of high-dose dexmedetomidine in critically ill patients. Crit Care.

[16] Zhang H, Zhou F, Li C (2013). Molecular mechanisms underlying the analgesic property of intrathecal dexmedetomidine and its neurotoxicity evaluation: an in vivo and in vitro experimental study. PLoS ONE.

[17] Gao YJ, Ji RR (2009). c-Fos and pERK, which is a better marker for neuronal activation and central sensitization after noxious stimulation and tissue injury?. Open Pain J.

[18] Kamal SM, Mohamed SA, Fares KM, Abdelemam RM, Elmasry HM, Mansour S (2022). Immunosuppressive Effect of Intrathecal Morphine, Dexmedetomidine, or Both in Combination with Bupivacaine on Patients Undergoing Major Abdominal Cancer Surgery. Pain Physician.

[19] Ozdamar D, Dayioglu H, Anik I, Solakoglu S, Solak M, Toker K (2018). Evaluation of the neurotoxicity of intrathecal dexmedetomidine on rat spinal cord (electromicroscopic observations). Saudi J Anaesth.

[20] Hou J, Xia Z, Xiao X, Wan X, Zhao B (2012). Neurotoxicity of intrathecal injections of dexmedetomidine into the rat spinal dorsal horn. Neural Regen Res.

[21] Liu S, Zhao P, Cui Y (2020). Effect of 5-μg Dose of Dexmedetomidine in Combination With Intrathecal Bupivacaine on Spinal Anesthesia: A Systematic Review and Meta-analysis. Clin Ther.

